# Bioinks of Natural Biomaterials for Printing Tissues

**DOI:** 10.3390/bioengineering10060705

**Published:** 2023-06-10

**Authors:** Girdhari Rijal

**Affiliations:** Department of Medical Laboratory Sciences, Public Health and Nutrition Science, Tarleton State University, a Member of Texas A & M University System, Fort Worth, TX 76104, USA; rijal@tarleton.edu

## 1. Introduction

Bioinks are inks—in other words, hydrogels—prepared from biomaterials with certain physiochemical properties together with cells to establish hierarchically complex biological 3D scaffolds through various 3D bioprinting technologies. The rheological, biological, chemical, and mechanical characteristics of biomaterials are considered the key physiochemical properties during the fabrication of a desired 3D tissue with specific cell types for both in vitro or in vivo studies. In addition, biomaterials should degrade over time, and cells should adjust, proliferate, differentiate, and grow in the host to establish normal anatomical and physiological functions. Ideally, bioinks can be prepared from any natural biomaterials based on their solubility in polar or nonpolar solvents. However, the proper rheological properties and optimum crosslinking mechanisms of bioinks are crucial in the fabrication of normal or tumor tissue models through bioprinting systems besides their biocompatibility, biodegradability, biomimicry, and mechanical properties.

Natural biomaterials are unique in their cell-binding sites and cross-linking properties (in the presence of UV light or certain enzymes or chemofactors) influencing the growth and differentiation of the cells [1,2]. The bioinks of some natural biomaterials are briefly discussed herein; details of the printing methodologies are beyond the scope of this article. Based on bioinks, a bioprinting system is adapted with by optimizing the procedures necessary for successful printing [3,4,5]. Printing is successful when the 3D-printed construct has high shape fidelity and the optimum microenvironment for cell survival, proliferation, and growth is assured [6,7]. Extrusion-based printing systems are very common in practice since they can print the construct from bioinks prepared from natural biomaterials such as alginate, gelatin, collagen, fibrin, gellan gum, hyaluronic acid (HA), agarose, chitosan, silk, decellularized extracellular matrix (dECM), and Matrigel [8,9]. Other alternate methods are laser-assisted cell printing, MHDS, inkjet bioprinting, rapid prototyping, robotic dispensing, droplet ejector printing, custom-made drop-on-demand bioprinting, dual syringe deposition, pneumatic dispensing, ED bioprinting, and valve ejector bioprinting.

## 2. Selection of Biomaterials for Bioinks

The selection of a bioink depends on its biocompatibility, printability, stability, and degradability, with no harm to the cells and inertness to the immune system [10,11]. The viscosity of a bioink is highly critical since it determines the shear stress, which affects the survivability of the suspended cells [12]. A high viscosity is better for fabricating tissue-mimicking constructs [13]. However, it is a challenge to maintain the viability of cells during bioprinting as they undergo additional shear stress due to the small nozzle involved in the process [14,15].

Bioinks, which are being introduced into regenerative medicine, drug delivery, and cancer research, are commonly classified as carbohydrate (polysaccharide)-based, protein-based, or complex (both carbohydrate and protein)-based; only a few of the more common ones are discussed here.

## 3. Carbohydrate-Based Bioinks

Alginate, agarose, carrageenan, cellulose, pectin, starch, dextran, xanthan gum, gellan gum, pullulan, and chitosan are carbohydrate-based natural biomaterials used to prepare bioinks. Only alginate, the most commonly used, is discussed here. It is a polysaccharide similar to the native human ECM polysaccharide, with high gelation and inertness and excellent biocompatibility, as well as adjustable viscosity, which is used to encapsulate the cells [16,17,18]. Because of its poor ability to bind cells, its modification is necessary; however, this can compromise its function. Antibacterial bioinks based on gelatin, alginate, and chitosan have recently been constructed for wound dressing [19]. In a similar vein, alginate can be composited with other types of biomaterials such as HA (for cartilage), laminarin–boronic acid and cholesteryl ester (for 3D constructs), and diethylaminomethyl cellulose and collagen (for drug screening) [20,21,22]. Extrusion-based printing, laser-assisted cell printing, MHDS, and inkjet bioprinting are common methods of bioprinting target tissues—mainly vascular tissues and tumoroids—using alginate.

## 4. Protein-Based Bioinks

For in vitro 3D models, protein-based printing bioinks are preferred because of their cross-linking ability and structural stability. They provide a more natural microenvironment for the growing cells compared to other nonproteinous natural bioinks. The majority of protein bioinks—for example, collagen, gelatin and dECM—are antigenic and trigger the recipient’s immune system, leading to the failure of the desired tissue (normal or cancerous) regeneration after an implant [23,24,25]. Collagens, particularly collagen type I, are highly abundant proteins found in the ECM. However, they have good thermosensitive and viscosity properties; these are not considered much as a suitable bioprinting material because of their weak gelation and rheological properties [26]. Laser-assisted bioprinting, robotic dispensing, droplet ejector printing, and custom-made drop-on-demand bioprinting are commonly used to fabricate skin, bone, and liver from collagen bioinks.

Gelatin is the product of denatured collagen that can be used as a thermosensitive bioink, but it is not recommended for high-fidelity scaffold fabrication because of its poor mechanical strength [27,28]. Cell assemblers, rapid prototyping with a 3D bioplotter, extrusion-based printing, and MHDS are bioprinting techniques which use gelatin to fabricate aortic valves, vascular tissue, and cartilage.

Fibrin is an inert, biodegradable, and biocompatible material able to induce cell attachment, proliferation, and support for ECM synthesis. It is a very common hydrogel in wound healing applications. It is, however, very challenging to establish printing constructs from fibrin because of its poor mechanical strength, non-crosslinking, and fast degradation properties [29,30]. Custom-made inkjet bioprinters and laser-assisted bioprinting are common methods of fabricating cartilage and vascular tissue from fibrinogen or fibrin.

## 5. Complex Bioinks

Complex bioinks have complex chemical structures made of both carbohydrate and proteins. HA, Matrigel, and dECM are discussed here briefly.

HA is a very soft and hydrating bioink as it contains glycosaminoglycan (GAG). HA, with its hydroxyl and carboxyl functional groups, is polymeric and polyelectrolytic in nature. Its high viscosity has less shear stress and vice versa. It possesses a negative charge in the physiological pH, influencing the biochemical and physiological properties of the ECM microenvironment [31]. HA itself is not suitable for printing constructs because of its instability and rapid degradation rate. However, with additional chemicals, for example methacrylate for cross-linking in the presence of UV rays, it can be designed as a printable bioink, but its natural properties are compromised [32,33]. Extrusion-based printing, commercial multimaterial bioprinters, inkjet bioprinting, pneumatic dispensing, ED heatable bioprinters with a temperature control unit and UV light source, and laser-assisted bioprinting systems are used to fabricate bone from HA.

Currently, the previously widely used Matrigel is not considered a natural bioink since it contains cancer-associated ECM proteins and other soluble proteins. Its use in bioprinting is still common in hybrid bioinks—most commonly with alginate for vascular tissue constructs [34,35,36]. It is not recommended for implants in human beings since it has hundreds of cancerous molecules that help divert normal tissue to cancerous ones [36,37]. However, pneumatic dispensing systems, extrusion-based bioprinting, laser-assisted bioprinting, and inkjet bioprinting use Matrigel for the fabrication of vascular tissues such as liver, bone, and lung.

Recently, dECM-based bioinks have been considered the front-line bioinks for tissue regeneration and tumor models [38,39]. The ECM components, percentile distribution, and assembly to maintain a suitable microenvironment are determined and tailored to the specific functional needs of the cells. Fibrous proteins, especially collagen and elastin, are the main frameworks for 3D ECM integrity. Some adhesion proteins, such as fibronectin and laminin, support the formation of ECM networks and are responsible for cell–ECM interactions. Certain other ECM proteins, such as glycoprotein and integrins, handle the cell–cell and cell–matrix interactions, guiding the cellular behavior. The ECM, therefore, not only provides a scaffolding system for the growing cells, but also delivers and relays signals through cell–cell or cell–ECM communications. It has been reported that if cells detach from the ECM scaffolding in their microenvironment, they cannot withstand for longer periods and undergo apoptosis or programmed death [40]. The physiological and metabolic function of the cells is also determined by the ECM type; for instance, fibroblasts proliferate faster in fibronectin than in laminin, whereas epithelial cells show the opposite phenomenon [41]. In addition, cells have the tendency to differentiate in interactions with specific ECM components. For example, through interacting with laminin, myogenic cells differentiate and form myotubes, but undergo no differentiation with fibronectin [42,43]. The percentile distribution of the different protein components and receptors in the ECM determines the role of different cell types present in the microenvironment that mediate cytoskeletal shapes [44]. Supporting cell migration is another important feature of the ECM. For example, fibronectin regulates fibroblast migration, while laminin regulates the migration of most cancer cells [45].

How the dECM is prepared also plays a significant role; there are physical, chemical, and enzymatic methods. Decellularization is considered complete if the DNA residue is not over 50 ng per mg of dECM dry weight, or the DNA fragment length is less than 200 base pairs [46]. The physical methods of decellularization change the cell membrane structure with the loss of mechanical strength of the ECM, switching to different biochemical reactions. The most common is freeze–thaw, where frequent temperature alteration leads to ECM damage [47]. Chemical methods, on the other hand, especially disrupt protein bonds among ECM proteins, which leads to the loss of the original configuration of the protein molecules, leading to different signature cascades through cell–ECM interactions [48]. Certain enzymes, for example pepsin, are used when preparing bioink to disrupt the bonds in the ECM, either with physical or chemical methods. The prolonged used of enzymes not only alters the chemical structures of the ECM proteins, but also degrades the structural proteins, for example collagen and its loss. In addition, residual enzymes, if present in the ECM bioink, could have adverse effects on the cells and the bioprinted scaffold itself [49]. The preparation of dECM without the use of pepsin is considered better for tissue engineering [50]. There are many dECM bioinks used in research labs and clinical trials, for example, photo-crosslinkable cartilage-derived dECM bioinks [51], cartilage-derived dECM [52] bioinks, liver-derived dECM bioinks [53], and skin-derived dECM bioinks [54].

dECM bioinks have inactive or active proteins, with the function of either stopping or relaying signals for particular influences on the cells. There is a high chance of altering or losing the natural configuration of protein molecules during the extraction, isolation, purification, and preparation of protein bioinks [55,56]. Non-native-state protein bioinks are incapable of providing a native-like microenvironment for cells and alter the cell signatures through matrix–cell interactions. Therefore, it is vital to prepare native-state protein bioinks to monitor tissue or tumor growth under controlled and guided systems to develop not only natural-like normal or abnormal tissue constructs, but to understand the underlying mechanisms of tissue growth. Bioprinting with plunger-based dispensing systems and extrusion-based bioprinting use dECM bioinks to fabricate adipose, heart, cartilage, and liver tissue.

## 6. Conclusions

In conclusion, for natural biomaterials, meeting the physiochemical requirements of each type of cell in their microenvironment and maintaining integrity with shape fidelity after bioprinting are the fundamental criteria to consider them as bioinks. The shear stress of a particular bioink and the tolerable capacity of each cell type highly influence the success of bioprinting. Changes in cell properties because of the physical and chemical nature of bioinks add more risks to obtaining the desired tissues. In the current trend, specific bioinks that provide suitable conditions for the survival and proliferation of a special cell type while keeping the native-like cellular properties are selected, which is not sufficient for mimicking the native tissue microenvironment. Therefore, optimizing bioinks that could represent the native-like ECM, suitable for all types of cells, is important for success in tissue engineering, drug delivery, cancer research, and personalized medicine.
